# Relationship Between the Estimated Breeding Values for Litter Traits at Birth and Ovarian and Embryonic Traits and Their Additive Genetic Variance in Gilts at 35 Days of Pregnancy

**DOI:** 10.3389/fgene.2018.00111

**Published:** 2018-04-05

**Authors:** Carolina L. A. Da Silva, Han A. Mulder, Marleen L. W. J. Broekhuijse, Bas Kemp, Nicoline M. Soede, Egbert F. Knol

**Affiliations:** ^1^Adaptation Physiology Group, Wageningen University and Research, Wageningen, Netherlands; ^2^Animal Breeding and Genomics, Wageningen University and Research, Wageningen, Netherlands; ^3^Topigs Norsvin Research Center B.V., Beuningen, Netherlands

**Keywords:** precision phenotyping, ovulation rate, corpora lutea weight, embryo, gilts

## Abstract

We investigated (1) the relationship between the estimated breeding values (EBVs) for litter traits at birth and ovulation rate (OR), average corpora luteal weight, uterine length and embryonic survival and development traits in gilts at 35 days of pregnancy by linear regression, (2) the genetic variance of OR, average corpora lutea (CL) weight, uterine length and embryonic survival and development traits at 35 days of pregnancy, and (3) the genetic correlations between these traits. Landrace (*n* = 86) and Yorkshire × Landrace (*n* = 304) gilts were inseminated and slaughtered at 35 days of pregnancy. OR was assessed by dissection of the CL on both ovaries. Individual CL was weighed and the average CL weight calculated. The number of embryos (total and vital) were counted and the vital embryos were individually weighed for calculation of within litter average and standard deviation (SD) of the embryo weight. Length of the uterine implantation site of the vital embryos was measured and the average per gilt calculated. Results suggests that increasing the EBV for total number of piglets born would proportionally increase OR and number of embryos, while decreasing the average CL weight. On the contrary, increasing the EBV for average piglet birth weight and for within litter birth weight standard deviation would increase the average CL weight. There was no relationship between the EBVs for BW and for BWSD and vital embryonic weight at 35 days of pregnancy. OR, average CL weight, number of embryos, average weight and implantation length of the vital embryos had all moderate to high heritabilities, ranging from 0.36 (±0.18) to 0.70 (±0.17). Thus, results indicate that there is ample genetic variation in OR, average CL weight and embryonic development traits. This knowledge could be used to optimize the balance between selection for litter size, average piglets birth weight and within litter birth weight uniformity.

## Introduction

Genetic selection for total number of piglets born (TNB) has been successful and more than 30 piglets weaned per sow per year can be achieved nowadays. However, genetic selection for sows ability to farrow a high number of piglets has led to a decrease in piglet mean birth weight (Quiniou et al., 2002), and an increase in within litter birth weight variation (Quiniou et al., 2002; Wolf et al., 2008). These negative associations between litter traits are partly genetic (Damgaard et al., 2003; Wolf et al., 2008), which makes it difficult to improve all traits simultaneously. It has been suggested that genetic selection for TNB has altered the balance between other litter component traits, specifically ovulation rate (OR) and uterine capacity, resulting in uterine crowding and compromised embryonic and fetal development (Père et al., 1997; Town et al., 2005; Foxcroft et al., 2006; Da Silva et al., 2016, 2017a). OR, the major genetic component of TNB (Schneider et al., 2014), has increased disproportionally due to genetic selection for TNB (Blasco et al., 1993), reaching averages of 25 up to 30 (Patterson et al., 2008; Da Silva et al., 2016). In sows, an increase in OR is related with a decrease in placental length of the vital embryos at 35 days of pregnancy (Da Silva et al., 2016), and in gilts an increase in OR is related with a higher variation in vital embryonic weight at 35 days of pregnancy (Da Silva et al., 2017a). This might compromise further fetal development leading to fetal mortality, but it might also lead to a lower average piglet BW and higher within litter BW variation. Therefore, knowledge about the underlying genetics of ovarian, uterine, and embryonic development characteristics might help understanding the mechanisms leading to litter characteristics at birth and the physiological consequences of genetic selection for litter traits at birth. Moreover, OR, embryonic survival and development traits are additional phenotypic traits that could be used to improve litter characteristics at birth by genetic selection. Thus, the objectives of this study were (1) to investigate the relationship between the EBVs for litter traits at birth and OR, average corpora lutea (CL) weight, uterine length and embryonic survival and development traits in gilts at 35 days of pregnancy, (2) to estimate the genetic variation of OR, average CL weight, uterine length and embryonic survival and development traits at 35 days of pregnancy, and (3) to estimate the genetic correlations between these traits.

## Materials and Methods

### Ethics and Approval Statement

The experiment and all measurements were approved by the Animal Welfare Committee of Wageningen University and Research in compliance with the Dutch Law on Animal Experimentation. The experiment was conducted between May and December 2016 at Wageningen University and Research (Wageningen, Netherlands).

### Animals and Housing

The study included a total of 390 pregnant gilts, from one farm, being 304 crossbred (C) gilts (Yorkshire × Landrace; Topigs Norsvin, Vught, Netherlands) and 86 purebred (P) Landrace gilts (Topigs Norsvin, Vught, Netherlands), which were used in 18 batches, one batch per week.

The gilts were group housed (six animals per 8 m^2^), with individual feeding stations and received liquid feeding. From weaning at day 25 till day 49 of age, gilts were fed a starter diet (9.68 NE MJ/kg, 9.13 g/kg of ileal digestible lysine), from day 50 up to day 105 gilts were fed a rearing diet (9.42 NE MJ/kg, 8.03 g/kg of ileal digestible lysine), and from day 106 until first insemination gilts were fed a second rearing diet (9.24 NE MJ/Kg, 7.35 g/kg of ileal digestible lysine). During the first 70 days the gilts were fed three times a day and from 71 days onward the gilts were fed twice a day. Gilts had free access to water at all times.

Gilts were inseminated at 248.4 ± 16.6 days (ranging from 212 to 292), one or two times with semen stored for 6.5 ± 1.6 days (ranging from 3 to 10 days). The semen was collected from 17 boars from the Tempo breeding line (Topigs Norsvin, Vught, Netherlands). The Tempo boar is bred from a Topigs Norsvin E-line (Large White type). Semen was processed at one Specific Pathogen Free (SPF) artificial insemination station (Varkens KI Nederland, Vught, Netherlands) and insemination doses of 1.2 billion cells per 80 ml were produced. Semen was stored and transported to the farm at 17°C ± 2°C.

The weight at first insemination for the P and C gilts was 165.6 ± 2.9 kg vs. 154.5 ± 2.3 kg, respectively (*P* ≤ 0.05), with an average back fat thickness of 14.0 ± 0.3 mm for P and 13.3 ± 0.2 mm for C gilts. Gilts were slaughtered at 34.7 ± 0.9 days of pregnancy (32 up to 37 days) with an average weight of 180.0 ± 15.5 kg.

### Ovarian, Embryonic, and Uterine Measurements

After slaughter, uterus and ovaries of the gilts were collected. OR was assessed by dissection of each individual corpus luteum present on left and right ovaries. After dissection, individual *corpus luteum* were cleaned of remaining connective tissue and individually weighed to assess average and standard deviation of CL weight (g). Total luteal mass was calculated as the sum of all CL weights.

Both uterine horns were separated from the mesometrium and opened at the anti-mesometrial side. After opening the uterus, embryos were classified as vital, non-vital or degenerated according to their visual appearance and were considered as non-vital when there was hemolyzed amniotic fluid, and degenerated when there were resorbed embryonic membranes or evidence of implantation, combined or not with placental or embryonic remnants (van der Waaij et al., 2010). After classification, embryos were separated from their placentas and counted. The total number of embryos was calculated as the sum of the vital embryos, non-vital embryos and of the degenerated embryos. The difference between OR and the total number of embryos was considered as early embryonic mortality, and the non-vital plus degenerated embryos were considered as late embryonic mortality.

The embryonic-placental units were separated from the uterine wall. After removal of the embryonic-placental units, implantation sites were identified by reddening of the endometrium, compared to the whiter area (unoccupied/empty uterine space) in between. The length and width of each implantation site on the uterine wall containing a vital embryo was measured and vital implantation area was calculated as the product of implantation length and implantation width. The empty uterine space around the vital embryos was measured and the average and standard deviation of the vital empty uterine space per gilt was estimated. The length of the left and right uterine horns were measured on a wet surface, from the utero-tubal junction to the uterine body. Total uterine length (cm) was measured as the sum of the left and right uterine horn length. In 254 of 390 gilts (batches 7–18), all vital embryos were individually weighed for assessment of average and standard deviation of vital embryonic weight (g).

### Relationship Between Estimated Breeding Values and the Phenotypic Traits

Analyses on the relationship between the estimated breeding values (EBVs) for gilts litter characteristics at birth and gilts ovarian, uterine, and embryonic characteristics at 35 days of pregnancy were performed using PROC MIXED in SAS 9.3 (SAS Inst. Inc., Cary, NC, United States). EBVs are best estimates of genetic merit. They come from the daily routine of Topigs Norsvin Research Center, where 17 reproductive traits are analyzed simultaneously in a multi-trait single step genomic BLUP evaluation (this includes genomic information for genotyped animals); data of 3 million sows and 10 million litters of a multitude of lines and crosses are used and corrected for known fixed effects as herd, year, season, and parity.

For clarification, an increase of one unit of EBV for TNB indicates an increase of one piglet phenotypically, an increase of one unit of EBV for BW indicates an increase of 1 kg in piglet birth weight, and an increase of one unit of EBV for BWSD indicates an increase of 1 g in within litter piglet birth weight standard deviation. Therefore, higher EBV for TNB and for BW indicates a higher genetic potential for higher litter size and higher average piglet birth weight, while higher EBV for BWSD indicates a higher variation and a lower genetic potential for within litter piglet birth weight uniformity.

Estimated breeding values for TNB (EBV_TNB), average piglet birth weight (EBV_BW), and within litter standard deviation of piglet birth weight (EBV_BWSD) were provided by Topigs Norsvin based on their routine genetic evaluation.

In all statistical models the effects of EBV_TNB, EBV_BW, and EBV_BWSD were fitted as linear regressions, together with the fixed class effect of genetic line to account for heterosis in the crossbred and a potential difference in genetic level between lines (GL, purebred Landrace, *n* = 86 and crossbred Yorkshire × Landrace, *n* = 304) and of semen storage duration classes (SS, SS_1_ = 3–5 days, *n* = 109; SS_2_ = 6–7 days, *n* = 159; and SS_3_ = 8–10 days, *n* = 122). The models also included the double interactions between EBVs, SS and GL, and the triple interactions between all model terms. In fact, interactions between EBVs and SS do not have biological meaning but may indicate some confounding, while interactions between EBVs and GL may imply difference in genetic variances between purebreds and crossbreds.

The fixed class effects and the interactions were excluded from the models when not significant. Fixed class effects of GL and SS were adjusted using Bonferroni. Residuals of all models were approximately normality distributed. Results were considered different at *P* ≤ 0.05 and are presented as regression coefficients (β) with their SE.

### Genetic Parameters

The following linear animal model was used for estimating variance components for the reproductive traits:

$$\begin{array}{c} Y_{ijx}=\mu +GL_{i}+SS_{j}+a_{x}+e_{ijx} \end{array}$$

where *Y_ijx_* are the reproductive traits [OR, average and standard deviation (SD) of CL weight, total luteal mass, uterine length, number of embryos, early and late embryonic mortality, average and SD of vital embryonic weight, of empty uterine space around the vital embryos, of vital embryos uterine implantation length, and area], μ is the overall mean, *GL_i_* is the fixed class effect of genetic line (86 Landrace and 304 Yorkshire × Landrace), *SS_j_* is the fixed class effect of semen storage duration classes (SS_1_ = 3–5 days, *n* = 109; SS_2_ = 6–7 days, *n* = 159 and SS_3_ = 8–10 days, *n* = 122), *a_x_* is the random additive genetic effect of the *x*th animal assumed to be ∼N(0, **A**$\sigma_{a}^{2}$), and *e_ijx_* is the residual term assumed to be ∼N(0, **I**$\sigma_{e}^{2}$). Assumed (co)variance structures of the random model terms are **A**$\sigma_{a}^{2}$, and **I**$\sigma_{e}^{2}$, in which **A** is the additive genetic relationship matrix, $\sigma_{a}^{2}$ is the additive genetic variance, **I** is an identity matrix, and $\sigma_{e}^{2}$ is the residual variance. The pedigree used to construct **A** consisted of 5,082 individuals, and was based on seven generations of ancestors.

Heritabilities were calculated as:

$$h^{2}=\frac{\sigma_{a}^{2}}{\sigma_{p}^{2}}$$

and phenotypic variance was $\sigma_{p}^{2}$ = $\sigma_{a}^{2}$ + $\sigma_{e}^{2}$, where $\sigma_{a}^{2}$ is the additive genetic variance, and $\sigma_{e}^{2}$ is the residual variance. Genetic correlations between traits were estimated using bivariate versions of the model given estimating both genetic correlations and residual correlations between traits. Phenotypic correlations were calculated by ASReml by summing the genetic and residual covariance divided by the phenotypic standard deviations of both traits. Fixed effects were tested for significance by an incremental Wald F statistics analysis (*P* ≤ 0.05).

## Results

Descriptive statistics are shown in **Table 1**. In total 390 pregnant gilts, of which 86 purebred Landrace and 304 crossbred Yorkshire × Landrace were phenotyped at 35 days of pregnancy for ovarian, uterine, and embryonic characteristics. Average OR was 20.7 ± 3.0, ranging from 14 up to 34; average CL weight was 0.44 ± 0.1 g, ranging from 0.27 to 0.61 g. Early embryonic mortality was on average 4.5 ± 4.3 and late embryonic mortality 1.2 ± 1.5. The number of vital embryos was 15.0 ± 4.1, with an average weight of 4.2 ± 0.8 g and an average uterine implantation length of 21.7 ± 4.3 cm. Within litter variation in vital embryo weight was 0.4 ± 0.1 g, ranging from 0.2 to 1.0 g and in uterine implantation length 5.4 ± 1.8, ranging from 2.1 to 29.2 cm.

**Table 1:** Summary statistics of phenotypic traits (ovarian, uterine, and embryonic characteristics) and of the estimated breeding values (EBV) of litter traits of gilts at 35 days of pregnancy.

Variables	*n*	Mean	*SD*	Min	Max
**Averages**					
Ovulation rate, *n*	390	20.7	3.0	14	34
Corpus luteum weight, g	390	0.44	0.06	0.27	0.61
Total luteal mass, g	390	9.1	1.6	5.3	16.4
Uterine length, cm	390	502.0	63.3	346	675
Number of embryos, *n*	390	16.2	4.3	3	26
Number of vital embryos, *n*	390	15.0	4.1	3	24
Early embryonic mortality, *n*	390	4.5	4.3	–2	21
Late embryonic mortality, *n*	390	1.2	1.5	0	9
Embryo weight, g	254	4.2	0.8	2.5	6.8
Empty space, cm	390	21.9	19.5	5.7	231.3
Implantation length, cm	390	21.7	4.3	11.27	38.4
Implantation area, cm^2^	390	192.7	44.8	77.4	397.8
**Standard deviations**					
Corpus luteum weight, g	390	0.05	0.02	0.02	0.13
Embryo weight, g	254	0.39	0.14	0.15	0.97
Empty space, cm	390	11.2	10.3	2.1	103.7
Implantation length, cm	390	5.4	1.8	2.1	29.2
**Estimated breeding values**					
Total number of piglets born, *n*	390	–0.09	0.61	–1.80	2.10
Average piglet birth weight, g	390	31.0	76.7	–245.6	282.3
Within litter birth weight variation, g	390	–0.04	15.7	–51.2	40.3

The EBV for TNB ranged from -1.80 to 2.10; for average piglet birth weight from -245.6 g to up to 282.3 g; and for within litter birth weight variation ranged from -51.2 g to up to 40.3 g. This shows that the genetic variance among the gilts in the experiment is substantial.

Effects of genetic line and semen storage duration classes on ovarian, uterine, and embryonic characteristics are at Supplementary Table S1.

### Relationship Between Estimated Breeding Values for Litter Traits and Ovarian, Uterine, and Embryonic Characteristics

The regression equations for the relationship between EBVs for TNB (EBV_TNB), average piglet birth weight (EBV_BW), and within litter birth weight standard deviation (EBV_BWSD) and ovarian, uterine, and embryonic characteristics of gilts at 35 days of pregnancy are presented in **Table 2**.

**Table 2:** Relationship between the gilts estimated breeding values for total number of piglets born (EBV_TNB), average piglet birth weight (EBV_BW) and within litter piglet birth weight standard deviation (EBV_BWSD) and their ovarian, uterine, and embryonic survival and development characteristics at 35 days of pregnancy.

Variables	EBV_TNB, *n*				EBV_BW, kg				EBV_BWSD, g			
	β ± SE	*P*-value	GL	SS	β ± SE	*P*-value	GL	SS	β ± SE	*P*-value	GL	SS
**Averages**												
Ovulation rate, *n*	1.12 0.2	**<0.0001**	**^∗∗^**	**–**	3.18 2.9	0.08	^∗∗^	**–**	0.05 0.01	0.62	**^∗∗^**	**–**
Corpus luteum weight, g	–0.011 0.004	**0.02**	ns	**–**	0.14 0.04	**0.0006**	ns	**–**	0.0006 0.0002	**0.003**	ns	**–**
Total luteal mass, g	0.27 0.12	**0.02**	**^∗∗^**	**–**	4.35 1.48	0.11	^∗∗^	**–**	0.024 0.01	**<0.0001^B^**	**^∗^**	**–**
Uterine length, cm	14.1 5.3	**0.001**	ns	ns	17.2 42.2	0.68	ns	ns	**–**0.20 0.2	0.34	ns	ns
Number of embryos, *n*	1.22 0.4	**0.001**	ns	ns	6.37 4.2	0.60	ns	ns	0.06 0.02	0.25	ns	**^∗∗^**
Number of vital embryos, *n*	1.12 0.3	**0.002**	ns	ns	5.72 3.9	0.69	ns	ns	0.05 0.03	0.76	ns	ns
Early embryonic mortality	–0.13 0.4	0.70	**^∗∗^**	**^∗∗^**	**–**0.17 2.9	0.95	^∗∗^	^∗∗^	0.01 0.01	0.33	**^∗∗^**	**^∗∗^**
Late embryonic mortality	0.10 0.1	0.42	ns	ns	**–**0.59 0.97	0.55	ns	ns	**–**0.004 0.01	0.44	ns	ns
Embryo weight, g	–0.05 0.1	0.41	**^∗^**	**^∗∗^**	**–**0.065 0.49	0.90	ns	^∗∗^	0.001 0.003	0.68	ns	**^∗∗^**
Empty space, cm	–3.3 1.6	**0.04**	ns	ns	**–**3.63 13.02	0.78	ns	ns	**–**0.25 0.1	0.52	ns	ns
Implantation length, cm	1.1 0.7	0.76	**^∗∗^**	ns	3.67 2.9	0.21	^∗^	ns	0.05 0.03	0.36	ns	ns
Implantation area, cm^2^	12.3 7.8	0.22	**^∗∗^**	ns	53.06 29.9	0.08	^∗∗^	ns	0.31 0.14	**0.03**	**^∗∗^**	ns
**Standard deviations**												
Corpus luteum weight, g	–0.003 0.002	0.07	ns	ns	0.0097 0.002	0.51	ns	ns	**–**0.00001 0.0001	0.94	ns	ns
Embryo weight, g	0.02 0.01	0.09	ns	ns	**–**1.62 0.46	0.58	ns	ns	**–**0.003 0.002	0.13	ns	ns
Empty space, cm	–1.9 0.9	**0.03**	ns	ns	2.34 7.02	0.74	ns	ns	**–**0.035 0.04	0.34	ns	ns
Implantation length, cm	0.21 0.3	0.52	ns	ns	0.75 2.2	**0.03^A^**	ns	ns	0.02 0.1	0.62	**^∗^**	**^∗^**

*Results come from three different models (one for each EBV). ns, not significant (*P* > 0.05); SE, standard errors; ^∗∗^*P* < 0.01; ^∗^*P* < 0.05. ^*A*^Interaction between semen storage duration classes and EBV_BW (*P* = 0.01): SS_*1*_ = 5.3 (0.1) + 7.5 (5.2)^∗^EBV_BW; SS_*2*_ = 5.3 (0.1) – 0.081 (0.49)^∗^EBV_BW; SS_*3*_ = 5.3 (0.1) + 0.75 (2.21)^∗^EBV_BW. ^*B*^Interaction between genetic line and EBV_BWSD (*P* = 0.01): Purebreds: 9.7 (0.4) + 0.005 (0.004)^∗^EBV_BWSD; Crossbreds: 9.0 (0.2) + 0.03 (0.01)^∗^EBV_BWSD. *P*-values of the significant relationships are shown in bold.*

An increase in the EBV_TNB was related with an increase in OR (β = 1.1 ± 0.2 CL/EBV) and with a decrease in average CL weight (β = -0.01 ± 0.004 g/EBV). Thus, a higher genetic potential for TNB is related with a higher OR but with a lower average CL weight.

An increase in the EBV_TNB was related with an increase in the total number of embryos (β = 1.2 ± 0.4 embryos/EBV) and in the number of vital embryos (β = 1.1 ± 0.3 embryos/EBV) in gilts at 35 days of pregnancy, but not with early and late embryonic mortality (*P* > 0.05). Thus, gilts with a higher genetic potential for TNB have a higher number of vital embryos at 35 days of pregnancy.

An increase in the EBV_TNB was related with an increase in uterine length (β = 14.1 ± 5.3 cm/EBV), and with a decrease in average empty uterine space (β = -3.3 ± 1.6 cm/EBV) and in variation in the empty uterine space (β = -1.9 ± 0.9 cm/EBV) around the vital embryos at 35 days of pregnancy. Thus, in gilts with a higher genetic potential for TNB there is less empty uterine space around the vital embryos, despite the higher uterine length at 35 days of pregnancy.

An increase in EBV_BW was related with an increase in the average CL weight (β = 0.14 ± 0.04 g/kg of EBV). Thus, gilts with a higher genetic potential for average piglet birth weight have a higher average CL weight at 35 days of pregnancy.

There was no relationship between the EBV_BW and the average and SD vital embryonic weight at 35 days of pregnancy. EBV_BW was also not related (*P* > 0.05) with the vital embryos average implantation length and area and empty uterine space. Thus, there is no relationship between gilts genetic potential for piglets birth weight and the weight of the vital embryos in early pregnancy.

An increase in EBV_BWSD was related with an increase in the average CL weight (β = 0.001 ± 0.0002 g/EBV). Thus, gilts with a higher genetic potential for within litter birth weight variation (i.e., lower uniformity) have a higher average CL weight at 35 days of pregnancy.

There was no relationship between the EBV_BWSD and average and SD of the vital embryonic weight, implantation length and empty uterine space at 35 days of pregnancy. There was, however, a positive relationship between EBV_BWSD and the vital embryos implantation area (β = 0.31 ± 0.14 cm^2^/EBV). Thus, a higher genetic potential for within litter piglet birth weight variation does not represent an increase in average or variation in weight of vital embryos at 35 days of pregnancy, but it seems to increase their implantation area at 35 days of pregnancy.

There were hardly any interactions between the EBVs and gilts genetic line and semen storage duration classes. An increase in EBV_BWSD was related with the total luteal mass in Yorkshire × Landrace gilts (+0.03 ± 0.01 g/g of EBV), but not in Landrace gilts (+0.005 ± 0.004 g/g). Thus, crossbred gilts with a higher genetic potential for within litter birth weight variation (i.e., lower uniformity) have a higher total luteal mass at 35 days of pregnancy.

It can be concluded that there is a strong positive relationship between EBV_TNB and the OR and number of vital embryos at 35 days of pregnancy, and between the EBVs for BW and BWSD and average CL weight.

### Heritabilities, Genetic, and Phenotypic Correlations

Heritabilities and additive genetic variance of ovarian, uterine, and embryonic traits are shown in **Table 3**. Genetic and phenotypic correlations between ovarian, uterine, and embryonic traits are presented in **Table 4**. Heritabilities and genetic correlations were considered as low when ≤|0.19| ; moderate when |0.20| to |0.40| and high when ≥|0.41|.

**Table 3:** Estimated variance components and heritabilities (± the standard errors) of ovarian, uterine, and embryonic characteristics in gilts at 35 days of pregnancy (*n* = 390).

Traits	Variance component^1^			
	$\sigma_{p}^{2}$	$\sigma_{a}^{2}$	$\sigma_{e}^{2}$	*h*^2^
**Averages**				
Ovulation rate, *n*	9.3 0.87	5.1 1.73	4.1 1.18	0.55 0.15
Corpus luteum weight, g	0.004 0.0004	0.0025 10^-3^	0.001 10^-3^	0.70 0.17
Total luteal mass, g	1.7 0.15	0.80 0.29	0.91 0.21	0.47 0.15
Uterine length, cm	4558.7 461.22	3098.9 962.60	1459.8 628.50	0.68 0.16
Number of embryos, *n*	19.4 1.72	8.1 3.25	11.2 2.39	0.42 0.14
Number of vital embryos, *n*	17.0 1.50	7.0 2.86	10.0 2.11	0.41 0.15
Early embryonic mortality, *n*	17.9 1.36	1.3 1.79	16.6 1.88	0.07 0.09
Late embryonic mortality, *n*	2.1 0.15	0.1 0.17	2.0 0.21	0.03 0.08
Embryo weight, g	0.36 0.04	0.13 0.07	0.23 0.06	0.36 0.18
Empty space, cm	383.3 30.37	73.0 46.24	310.3 41.55	0.19 0.11
Implantation length, cm	19.6 1.77	8.7 3.45	10.9 2.50	0.44 0.15
Implantation area, cm^2^	2101.1 201.46	1162.0 411.92	939.5 282.74	0.55 0.16
**Standard deviations**				
Corpus luteum weight, g	0.001 0.00003	0.00001 0.00002	0.001 0.00004	0.01 0.06
Embryo weight, g	0.02 0.002	0.0 0.002	0.02 0.002	0.002 0.09
Empty space, cm	108.8 9.21	29.9 17.12	78.8 13.72	0.28 0.14
Implantation length, cm	3.2 0.24	0.22 0.29	3.02 0.32	0.07 0.09

*^*1*^$\sigma_{p}^{2}$ phenotypic variance, $\sigma_{a}^{2}$ additive genetic variance, $\sigma_{e}^{2}$ residual variance.*

**Table 4:** Estimated genetic correlations (*in italic below the diagonal*) and phenotypic correlations (above the diagonal) of ovarian, uterine, and embryonic characteristics in gilts at 35 days of pregnancy (*n* = 390).

Traits^1^	OR	CL	LM	UL	E	VE	VEW	VES	VIL	VIA	SDVES
OR, *n*	**–**	**–**0.47 ± 0.05	0.58 ± 0.05	0.30 ± 0.06	0.40 ± 0.05	0.33 ± 0.06	0.04 ± 0.08	**–**0.17 ± 0.06	**–**0.13 ± 0.06	**–**0.11 ± 0.07	**–**0.12 ± 0.06
CL, g	**–***0.58 ± 0.16*	**–**	0.48 ± 0.05	**–**0.08 ± 0.07	**–**0.24 ± 0.06	**–**0.23 ± 0.06	0.17 ± 0.08	0.11 ± 0.06	0.24 ± 0.06	0.23 ± 0.07	0.15 ± 0.07
LM, g	*0.53 ± 0.18*	*0.42 ± 0.20*	**–**	0.20 ± 0.07	0.20 ± 0.06	0.14 ± 0.06	0.16 ± 0.08	**–**0.14 ± 0.06	0.02 ± 0.06	0.05 ± 0.07	**–**0.05 ± 0.06
UL, cm	*0.45 ± 0.20*	**–***0.37 ± 0.21*	*0.12 ± 0.24*	**–**	0.33 ± 0.06	0.38 ± 0.06	0.22 ± 0.07	**–**0.11 ± 0.06	0.35 ± 0.06	0.31 ± 0.06	**–**0.08 ± 0.06
E, *n*	*0.93 ± 0.12*	**–***0.88 ± 0.12*	*0.03 ± 0.27*	*0.49 ± 0.21*	**–**	0.95 ± 0.01	**–**0.05 ± 0.07	**–**0.76 ± 0.02	**–**0.59 ± 0.04	**–**0.40 ± 0.05	**–**0.66 ± 0.03
VE, *n*	*0.85 ± 0.15*	**–***0.87 ± 0.11*	**–***0.03 ± 0.27*	*0.58 ± 0.19*	*0.99 ± 0.01*	**–**	**–**0.06 ± 0.07	**–**0.74 ± 0.03	**–**0.51 ± 0.04	**–**0.31 ± 0.06	**–**0.64 ± 0.03
VEW, g	**–***0.03 ± 0.32*	**–***0.01 ± 0.34*	**–***0.05 ± 0.36*	*0.23 ± 0.30*	**–***0.08 ± 0.35*	*0.01 ± 0.35*	**–**	0.05 ± 0.07	0.25 ± 0.07	0.20 ± 0.07	0.09 ± 0.07
VES, cm	**–***0.71 ± 0.29*	*0.86 ± 0.17*	*0.11 ± 0.33*	**–***0.48 ± 0.30*	**–***0.84 ± 0.13*	**–***0.86 ± 0.13*	*0.14 ± 0.46*	**–**	0.43 ± 0.05	0.23 ± 0.1	0.72 ± 0.03
VIL, cm	**–***0.29 ± 0.24*	*0.64 ± 0.19*	*0.27 ± 0.27*	*0.56 ± 0.19*	**–***0.31 ± 0.24*	**–***0.22 ± 0.26*	*0.19 ± 0.33*	*0.13 ± 0.36*	**–**	0.83 ± 0.02	0.40 ± 0.05
VIA, cm^2^	**–***0.34 ± 0.22*	*0.58 ± 0.18*	*0.22 ± 0.26*	*0.27 ± 0.22*	**–***0.42 ± 0.22*	**–***0.29 ± 0.25*	*0.31 ± 0.29*	*0.12 ± 0.35*	*0.88 ± 0.07*	**–**	0.28 ± 0.06
SDVES, cm	**–***0.59 ± 0.27*	*0.93 ± 0.11*	0.38 ± 0.28	**–***0.37 ± 0.30*	**–***0.92 ± 0.12*	**–***0.98 ± 0.14*	*0.11 ± 0.42*	*0.80 ± 0.17*	*0.41 ± 0.28*	*0.49 ± 0.25*	**–**

*Heritabilities and genetic correlations were considered as low when ≤|0.19| ; moderate when |0.20| to |0.40| and high when ≥|0.41|. ^*1*^OR, ovulation rate; CL, corpus luteum weight (g); LM, luteal mass (sum of all corpora lutea weight, g); UL, total uterine length (cm); E, total number of embryos; VE, number of vital embryos; VEW, average individual vital embryo weight (g); VES, average empty uterine space around the vital embryos; VIL, average length of vital embryos uterine implantation (cm); VIA, average area of vital embryos uterine implantation (length × width of the implantation, cm^*2*^); SDVES, within litter variation in empty uterine space around the vital embryos.*

Heritability estimates were high for OR, average CL weight, total luteal mass, uterine length, number of embryos (vital and total) and vital implantation length and area; moderate for average vital embryonic weight at 35 days of pregnancy, and average and standard deviation of vital empty space around the vital embryos; and low for early and late embryonic mortality, and the SD of CL weight, SD of vital embryo weight, and the SD of vital empty space around the vital embryos. The genetic correlations between the ovarian uterine and embryonic number and development traits were in 50% of the cases high (≥0.41) and in approximately 24% of the cases moderate (0.20–0.40) For the traits with low heritabilities (≤0.19), genetic and phenotypic correlations were not estimated, because of convergence issues with ASReml or extremely high standard errors.

High positive genetic correlations included the correlations between OR and total luteal mass, uterine length and the number of embryos (total and vital), between average CL weight and total luteal mass, between uterine length and number of embryos (total and vital) and the average implantation length. In other words, all traits related to numbers or total mass or length are highly positively correlated indicating that these traits are partly controlled by the same genes.

High negative genetic correlations included the correlations between OR and average CL weight, average and SD of empty uterine space around the vital embryos, between the average CL weight and the number of embryos (total and vital), the average and SD of vital empty space around the vital embryos, and the average implantation length and area. In other words, higher OR or higher number of vital embryos are genetically associated with lower CL weight, average and SD of empty uterine space and average implantation length and area, which shows a trade-off between number of embryos and weight and space per embryo.

Results show that related traits such as OR and number embryos are partly controlled by the same genes as expected because of their dependencies, whereas a negative trade-off was observed between OR and number of embryos with CL weight and space per embryo.

## Discussion

We investigated how genetic selection based on the EBVs for litter traits at birth is related with ovarian, uterine, and embryonic traits in gilts at 35 days of pregnancy and estimated the additive genetic variance of these underlying traits. To our knowledge this is the first study to estimate the relationship between the genetic potential for litter traits at birth and ovarian and embryonic traits. It is also the first study to estimate the additive genetic variance of corpora luteal weight and embryonic survival and development traits.

Litter traits at birth are genetically and phenotypically negatively correlated, and genetic selection for a higher TNB seems to have simultaneously compromised the average piglet birth weight and within litter birth weight uniformity (Milligan et al., 2002; Damgaard et al., 2003; Wolf et al., 2008). Litter traits are composite traits, being dependent on the interactions between several underlying traits, such as OR, embryonic survival and development and uterine capacity (Johnson et al., 1984). It is important to know how these underlying traits are related with genetic improvement of litter traits at birth and if these traits are heritable, as this information could be used in genetic selection programs for litter traits at birth.

### Relationship Between EBVs and Traits at 35 Days of Pregnancy

Ovulation rate is genetic correlated with the TNB (Haley and Lee, 1993; Johnson et al., 1999; Rosendo et al., 2007), and is considered as the main component trait of litter size (Schneider et al., 2014). In this study population, an increase of one unit of EBV for TNB was related with an increase of one unit of OR (i.e., one more corpus luteum). This linear increase in OR of about 1 oocyte per unit of EBV_TNB is surprising, because in practice OR seems much higher than TNB and shows more variation than TNB. Therefore, a higher regression coefficient than 1 would have been expected, but was not found. One reason might be that current selection programs are more balanced by selecting simultaneously on TNB, birth weight and birth weight uniformity.

Surprisingly, we did not observe an increase in embryonic mortality with the increase in EBV for TNB, which was, on the other hand, related with a close to unity increase in the number of vital embryos. Thus, results suggest that limitations in the phenotypic response in the TNB with the increase in OR are probably established after 35 days of pregnancy.

Genetic selection for a higher TNB has been associated with an increase in uterine crowding (Bérard et al., 2010), which increases the competition between littermates for adequate uterine space for placental development, which has negative consequences for embryonic survival and development. However, an increase in the EBV for TNB did not influence uterine implantation length and area of the vital embryos, both important traits for placental development (Stroband and Van der Lende, 1990), or the weight of the vital embryos at 35 days of pregnancy. On the contrary, an increase in the EBV for TNB was related with an increase in uterine length, which suggests that gilts with a higher EBV for TNB do not have compromised development of the vital embryos due to a higher uterine crowding. Thus, a higher genetic potential for TNB is not related with a compromised development of the vital embryos at 35 days of pregnancy.

Although gilts with a higher EBV for TNB did not have compromised embryonic development up to 35 days of pregnancy they had a lower average CL weight at 35 days of pregnancy. Smaller CL indicates smaller follicles at ovulation (Wientjes et al., 2012). Smaller follicles at ovulation might release oocytes with lower quality, leading to the development of embryos with lower quality and reduced potential to grow (Ding and Foxcroft, 1994; Gandolfi et al., 2005). Thus, a higher genetic potential for TNB, might lead to compromised fetal development (i.e., after day 35 of pregnancy) due to a compromised follicular and oocyte quality, as indicated by the lower average CL weight.

Another indication of the importance of CL size for embryonic and fetal development, is the surprising absence of an association between the EBV for BW and uterine length, empty uterine space and measurements of embryonic development at 35 days of pregnancy (average implantation length and area, and weight of the vital embryos), while being significantly related with average CL weight. At the phenotypic level an increase of 1 g in the average weight of the CL is related with an increase of 2.3 g in the average weight of the vital embryos at 35 days of pregnancy for the gilts in this study (*P* = 0.001, *results not shown*). In multiparous sows at ∼3weeks of pregnancy with an average CL diameter ranging from 9.0 to 10.5 mm, average piglet birth weight was 1338.3 ± 27.3 g, while in sows with an average CL diameter ranging from 5.5 to 7.8 mm average piglet birth weight was 1270.5 ± 30.9 g, independent of the litter size they were born in (*P* < 0.05; Da Silva et al., 2017b).

There are three possible explanations for the positive relationship between CL size and piglet birth weight. Firstly, as discussed above, CL size might indicate the release of oocytes with higher quality (Marchal et al., 2002), which might favor early embryonic development. At days 10 to 11 of pregnancy, pig conceptuses start to elongate (Geisert et al., 1982). and timing of rapid elongation is established by the conceptuses (Geisert et al., 2014). Conceptuses with higher development potential might elongate earlier and quicker, taking up more uterine space (i.e., uterine implantation length) (Vallet et al., 2009). The length of the implantation site determines the length of the placenta (Stroband and Van der Lende, 1990). Thus, larger CL might be associated with embryos with higher quality, that acquire a larger placenta being therefore heavier at 35 days of pregnancy. Heavier embryos at 35 days of pregnancy are likely to develop into heavier piglets at birth, since most of the eventual weight of the embryos and its placentas are established up to 35 days of pregnancy (Vallet et al., 2009). Secondly, since CL produce progesterone, heavier CL could produce higher amounts of progesterone, favoring embryonic growth and piglet birth weight. However, systemic progesterone levels analyzed for a subset of 238 gilts at 35 days of pregnancy were not related (*P* = 0.69) with average CL weight (*results not shown*). Thirdly, CL development and embryonic development may share a common origin. During elongation conceptuses produce 17β-oestradiol (E2), which increases the expression of luteinizing hormone (LH) receptor in the luteal cells (Ziecik et al., 2011). LH, together with interleukin β1, favors the production of prostaglandin E2 by the CL, a potent luteoprotective that increases luteal permeability and delivery of cholesterol to the luteal cells by stimulating the expression of vascular endothelial growth factor (Ziecik et al., 2011), which might increase CL growth. Thus, the relationship between EBV for BW and average CL weight and not with any trait regarding vital embryonic development at 35 days of pregnancy, indicates that genetic improvement in piglet birth weight might occur through improvements in follicular and oocyte quality.

Similarly to what was observed for EBV for BW, an increase in the EBV for BWSD was not related with uterine length, empty uterine space around the vital embryos and also not with most of the measurements of development of the vital embryos at 35 days of pregnancy. An increase in EBV for BWSD was related with an increase in average CL weight. As a higher EBV for BWSD indicates gilts with a higher within litter piglet birth weight variation, genetic improvement in piglet birth weight uniformity occurs through a decrease in EBV for BWSD. Within litter variation (SD) in piglet birth weight is genetically positively correlated with average piglet birth weight (Damgaard et al., 2003; Sell-Kubiak et al., 2015), and consequently the EBVs for BW and BWSD are correlated (*r* = 0.69, *P* < 0.0001, *this study, results not shown*). This correlation can also explain the higher average CL weight in gilts with higher EBV for BWSD. Phenotypically, multiparous sows at ∼3weeks of pregnancy with an average CL diameter ranging from 9.0 to 10.5 mm had not only a higher average piglet BW but also a higher within litter piglet birth weight variation (318.6 ± 17.0 g) than sows with an average CL diameter ranging from 5.5 to 7.8 mm (252.2 ± 17.9 g, *P* < 0.05; Da Silva et al., 2017b). Thus, the increase in average CL weight in gilts with a higher genetic potential for within litter piglet birth weight variation might be a consequence of the genetic association between BW and BWSD.

The genetic association between BW and BWSD might also explain the higher variation in implantation length observed in gilts with a higher EBV for BW. The length of uterine implantation is a consequence of the length of embryonic elongation at earlier stages of pregnancy (Geisert et al., 1982). So, a higher variation in implantation length indicates a higher variation in embryonic elongation length before implantation. A higher variation in implantation length will lead to a higher variation in placental length (Stroband and Van der Lende, 1990), which might lead to a higher variation in fetal growth and in piglet birth weight. However, this should be interpreted carefully due to the high standard error of the beta. Thus, it is possible that gilts with a higher EBV for BW might have a higher piglet birth weight variation due to a higher variation in uterine implantation length and consequently in placenta growth at early pregnancy.

### Heritabilities and Genetic Correlations

Ovarian, uterine, and embryonic characteristics in gilts at 35 days of pregnancy are underlying traits influencing litter characteristics at birth. Results indicate that these traits are highly heritable and could be used to improve the accuracy of genetic selection programs for litter traits at birth. However, results should be interpreted carefully because the study population was small resulting in high standard errors. Furthermore, heritabilities and genetic correlations might be biased because the traits under selection, i.e., TNB, BW, and BWSD, were not included in the analysis (Meyer and Thompson, 1984; Sorensen and Johansson, 1992). Nevertheless, the used gilts were not selected and restricted maximum likelihood with an animal model was used limiting the potential bias in the estimated genetic parameters. OR is known to be heritable, and in this study 55% of its phenotypic variance was explained by genetic variance. Previous studies on the heritability of OR reported values of 0.27 in gilts at 27–30 days of pregnancy (Bidanel et al., 1996), 0.24 in gilts at 50 days of pregnancy (Johnson et al., 1999) and, more recently, 0.32 in a genome wide association study (Schneider et al., 2014). Despite the higher heritability in this study in comparison with the reports on literature, the additive genetic variance of OR in the present study was (5.1) similar to the genetic variance observed by Johnson et al. (1999) (6.6) and Schneider et al. (2014) (4.9). This suggests that the higher heritability of OR observed in this study is related with a lower residual or environmental variance, since it was based on a very homogeneous dataset (i.e., only gilts from a single farm), and on precise phenotyping (estimations were done by dissection of ovaries and counting individual CL, which is the most precise method for estimations of OR). This can also explain the high heritabilities described for other traits in this study.

Average CL weight and total luteal mass were also highly heritable. Phenotypically, an increase in OR is related with a decrease in average CL weight and an increase in total luteal mass in gilts (Da Silva et al., 2017a). These relationships are at least partly genetic, as OR had a negative genetic correlation with average CL weight, and a positive genetic correlation with total luteal mass. This indicates that genetic selection to increase OR might simultaneously select for a decrease in average CL weight, which could compromise embryonic weight at 35 days of pregnancy and piglet BW.

Heritability for the number of embryos (total and vital) at 35 days of pregnancy were also higher (0.42 and 0.41, respectively) than previously reported in literature: a heritability estimate of 0.14 for number of embryos at 30 days of pregnancy (Bidanel et al., 1996) and a heritability estimate of 0.18 for number of fetuses at day 50 of pregnancy (Johnson et al., 1999), but are still within the range of heritability described for TNB (up to 0.76) and number of piglets born alive (up to 0.66) (Bidanel, 2011). This indicates that it is possible to improve the number of vital embryos at 35 days of pregnancy, although it is not known whether it would improve the number of piglets born alive.

Early and late embryonic mortality had low heritability (0.07 and 0.03, respectively) similarly to what has been previously reported for embryonic survival up to 30 days of pregnancy (Bidanel et al., 1996). The low additive genetic variance of early embryonic mortality suggests that this trait is hardly under genetic control. For instance, early embryonic mortality is estimated as the difference between OR and the total number of embryos in the uterus at 35 days of pregnancy, and assumes an optimal fertilization rate (∼100%), which is normally achieved with the use of fresh semen (<24 h of storage) (De Ambrogi et al., 2006). However, in this study, gilts were inseminated with semen stored for 3 up to 10 days, and a decrease in fertilization rate might have led to an overestimation in early embryonic mortality. Regarding late embryonic mortality, genetic selection to improve it might not be possible, considering that this trait has an additive genetic variance close to zero. Thus, early and late embryonic mortality seem to be mainly influenced by environment factors.

Average implantation length and area of the vital embryos at 35 days of pregnancy had high heritabilities. As the length and area of uterine implantation determines the size of the placenta (Stroband and Van der Lende, 1990), improvement on these traits could benefit embryonic and fetal growth and consequently piglet birth weight. Also, the average weight of the vital embryos at 35 days of pregnancy had a heritability similar to what has been described for piglet birth weight by Knol (2001) (0.30) and by Damgaard et al. (2003) (0.39). Thus, vital embryonic development could be improved through genetic selection, and could be used to improve piglet birth weight.

Moreover, both uterine length and the empty uterine space around the vital embryos were heritable traits and could be used to alleviate the incidence of uterine crowding at 35 days of pregnancy through genetic selection. Although uterine length at 35 days of pregnancy is influenced by the number of embryos (Wu et al., 1987), it had a higher heritability than the uterine length in prepuberal gilts (0.50; Young et al., 1996). Thus, both vital embryonic development and the uterine space for such development up to 35 days of pregnancy could be improved through genetic selection, and could lead to an increase in piglet birth weight.

To use ovarian, uterine, and embryonic survival and development traits at 35 days of pregnancy in genetic selection programs, it is important to understand how selecting for one trait might influence other traits genetically (i.e., correlated responses). Phenotypically, an increase in OR is related with an increase in uterine length at 35 days of pregnancy (Da Silva et al., 2016, 2017a) and with an increase in the number of embryos at early pregnancy (Vonnahme et al., 2002; van der Waaij et al., 2010; Da Silva et al., 2016, 2017a). Moreover, an increase in OR is related with a decrease in the average implantation length (Da Silva et al., 2016, 2017a), in placental length and in average empty uterine space around the vital embryos at 35 days of pregnancy (Da Silva et al., 2016). These relationships are at least partly genetic, as the genetic correlations between these traits were similar to the phenotypic correlations above described. This indicates that, genetic selection for a higher OR might simultaneously compromise vital embryonic development at 35 days of pregnancy, which might lead to a decrease in average piglet birth weight. Moreover, results indicate that genetic improvement in CL weight at early pregnancy, might select for gilts with embryos with a better development potential, leading to piglets with a higher average birth weight. However, it is important to consider that average CL weight has strong negative genetic correlations with OR (-0.58), uterine length (-0.37) and number of embryos (-0.88) at 35 days of pregnancy. Thus, genetic improvement of these traits simultaneously might be difficult, and development of a genetic selection index balancing these traits would be necessary.

The negative associations between litter traits at birth are partly genetic (Damgaard et al., 2003; Wolf et al., 2008). Although at 35 days of pregnancy, the genetic correlations between the number of embryos (total and vital) and the average vital embryonic weight was close to zero, it was negatively genetically correlated with the average uterine implantation length and area. This indicates that, the negative genetic associations between litter size and birth weight are not yet present at early pregnancy in this population, but it might originate in later pregnancy, as fetal development might be compromised in gilts with higher number of embryos.

Thus, genetic improvement in TNB, average piglet birth weight and within litter piglet birth weight variation is mainly related with OR and average CL weight in gilts at 35 days of pregnancy. This study also provides genetic parameters estimates of component traits of litter characteristics at birth, although the study population is small. It confirms the existence of additive genetic variance for OR, and also indicates the existence of additive genetic variance for average CL weight, a trait that has been phenotypically related with vital embryonic weight at 35 days of pregnancy. Moreover, uterine and embryonic development traits at 35 days of pregnancy are also heritable. This gives opportunity to include precise phenotyping in genetic selection programs, in order to minimize the undesired associations between litter traits at birth. The traits OR and average CL weight seem to be the best two candidate traits to be measured on female selection candidates and their relatives and to be included in genetic selection programs. Larger datasets need to provide more reliable estimates of genetic parameters to evaluate the usefulness in practical breeding programs. Moreover, future genome wide association studies might help unraveling genetic variation of the underlying traits influencing litter characteristics at birth and may help to improve accuracy of genomic prediction.

## Author Contributions

CDS performed the research, analyzed and interpreted the data, and wrote the manuscript. HM helped in the data analyses and in the interpretation of the results. MB helped in the data collection and in the interpretation of the results. BK and NS helped in the data interpretation. EK designed the research and interpreted the data. All authors contributed to the final manuscript.

## Conflict of Interest Statement

This study was in kind funded by Topigs Norsvin. In addition, EK and MB are employed by Topigs Norsvin Research Center B.V. MB was involved in the data collection, and both EK and MB contributed to the interpretation of the results. However, the possible conflict of interest did not interfere with the outcome of this paper. The other authors declare that the research was conducted in the absence of any commercial or financial relationships that could be construed as a potential conflict of interest.
