# Supplementary material for: Relationship Between the Estimated Breeding Values for Litter Traits at Birth and Ovarian and Embryonic Traits and Their Additive Genetic Variance in Gilts at 35 Days of Pregnancy
Source: Front Genet. 2018 Apr 5;9:111. doi: 10.3389/fgene.2018.00111 (PMC5896267; doi:10.3389/fgene.2018.00111)
Supplement: Supplementary file 1 [file Table_1.pdf]

Supplementary Material

Consequences Of Genetic Selection For Litter Traits At Birth On Ovarian And Embryonic Traits In Gilts And Their Genetic Background

Carolina L.A. Da Silva[1*], Han A. Mulder[2], Marleen L.W.J. Broekhuijse[3], Bas Kemp[1], Nicoline M. Soede[1], Egbert F. Knol[3]

*** Correspondence: Carolina L.A. Da Silva** Corresponding Author: carolina.lima@wur.nl

Supplementary Table S1 . Least square means and SEM for ovarian and embryonic characteristics of gilts at 35 days of pregnancy for two different genetic lines (GL, Purebred Landrace and Crossbred Yorkshire x Landrace, respectively) and three different semen storage duration classes (SS; 3-5, 6-7 and 8-10 days, respectively). Batch (n=18) was included in the models as a random effect.

**Variables**	**Genetic Line (GL)**		**Semen Storage Classes (SS, d)**			**P Values**		
	**P**	**C**	**3 -5**	**6-7**	**8-10**	**GL**	**SS**	**GL*SS**
n=	86	304	109	159	122			
Averages								
Ovulation Rate, OR	22.10±0.35	20.31±0.19	21.06±0.32	21.13±0.27	21.42±0.31	**<.0001**	0.62	NS
Corpus luteum weight, g	0.44±0.01	0.44±0.01	0.44±0.01	0.45±0.01	0.44±0.01	0.68	0.26	NS
Total luteal mass, g	9.68±0.3	8.97±0.17	9.26±0.22	9.41±0.20	9.30±0.21	**0.006**	0.74	NS
Uterine length, cm	493.40±7.6	504.25±4.2	504.71±6.9	503.13±5.8	488.63±6.6	0.20	0.09	NS
Number of embryos [1]	15.61±0.5	16.33±0.3	15.93±0.5	16.56±0.4	15.42±0.5	0.18	0.18	**0.008**
Number of vital embryos [2]	14.46±0.5	15.13±0.2	14.875±0.5	15.20±0.4	14.32±0.5	0.18	0.34	**0.004**
Early embryonic mortality	6.18±0.5	4.03±0.2	4.30±0.4a	4.84±0.4a	6.17±0.4b	**<.0001**	**0.002**	NS
Late embryonic mortality	1.19±0.2	1.20±0.1	1.16±0.2	1.36±0.1	1.07±0.2	0.98	0.25	NS
Embryo weight, g	4.39±0.2	4.16±0.1	4.44±0.2a	4.31±0.1a	4.06±0.2b	**0.05**	**0.002**	NS
Empty space [3], cm	23.44±2.3	21.46±1.2	22.58±2.3	20.95±1.8	23.81±2.3	0.44	0.60	**0.03**
Implantation length, cm	22.86±0.5	21.30±0.2	21.85±0.4	22.18±0.4	22.21±0.4	**0.003**	0.77	NS
Implantation area, cm^2	213.17±4.8	187.56±2.5	204.2±4.5	196.7±3.7	200.2±4.3	**<.0001**	0.39	NS
Standard Deviations								
Corpus luteum weight, g	0.05±0.003	0.05±0.002	0.05±0.002	0.05±0.002	0.05±0.002	0.47	0.08	NS
Embryo weight, g	0.42±0.02	0.39±0.02	0.40±0.02	0.39±0.02	0.42±0.02	0.21	0.46	NS
Empty space [4], cm	11.58±1.1	11.05±0.6	11.51±1.2	10.71±0.9	14.40±2.1	0.68	0.76	**0.009**
Implantation length [5], cm	5.78±0.2	5.31±0.1	5.94±0.2	5.30±0.2	5.41±0.2	**0.05**	0.06	**0.03**

[1] Least square means estimates for the interactions genetic line*semen storage duration (P=0.008): Purebreds*SS1=14.4±0.9a; Purebreds*SS2=17.0±0.7ab; Purebreds* SS3=15.5±0.9ab; Crossbreds*SS1=17.5±0.5b; Crossbreds*SS2=16.2±0.4ab; Crossbreds*SS3=15.3±0.4a.

[2] Least square means estimates for the interactions genetic line*semen storage duration (P=0.008): Purebreds*SS1=13.4±0.8a; Purebreds*SS2=15.6±0.6ab; Purebreds* SS3=14.4±0.9ab; Crossbreds*SS1=16.4±0.4b; Crossbreds*SS2=14.5±0.4ab; Crossbreds*SS3=14.2±0.4a.

[3] Least square means estimates for the interactions genetic line*semen storage duration (P=0.03): Purebreds*SS1=27.9±4.1ab; Purebreds*SS2=18.4±3.1ab; Purebreds* SS3=24.0±4.2ab; Crossbreds*SS1=17.3±2.2a; Crossbreds*SS2=23.5±1.8b; Crossbreds*SS3=23.6±1.9b.

[4] Least square means estimates for the interactions genetic line*semen storage duration (P=0.01): Purebreds*SS1=14.4±2.1a; Purebreds*SS2=8.98±1.6b; Purebreds* SS3=11.4±2.2ab; Crossbreds*SS1=8.6±1.1b; Crossbreds*SS2=12.4±0.9a; Crossbreds*SS3=12.1±1.0a.

[5] Least square means estimates for the interactions genetic line*semen storage duration (P=0.03): Purebreds*SS1=6.59±0.4a; Purebreds*SS2=5.26±0.3ab; Purebreds* SS3=5.50±0.4ab; Crossbreds*SS1=5.28±0.2ab; Crossbreds*SS2=5.35±0.2ab; Crossbreds*SS3=5.31±0.2b.
